# Perception of extreme hot weather and the corresponding adaptations among older adults and service providers–A qualitative study in Hong Kong

**DOI:** 10.3389/fpubh.2023.1056800

**Published:** 2023-02-10

**Authors:** Eric T. C. Lai, Pui Hing Chau, Ken Cheung, Michelle Kwan, Kevin Lau, Jean Woo

**Affiliations:** ^1^Institute of Health Equity, The Chinese University of Hong Kong, Hong Kong, Hong Kong SAR, China; ^2^Department of Medicine and Therapeutics, Faculty of Medicine, The Chinese University of Hong Kong, Hong Kong, Hong Kong SAR, China; ^3^School of Nursing, LKS Faculty of Medicine, The University of Hong Kong, Hong Kong, Hong Kong SAR, China; ^4^Jockey Club Institute of Ageing, The Chinese University of Hong Kong, Hong Kong, Hong Kong SAR, China; ^5^Department of Civil, Environmental and Natural Resources Engineering, Luleå Tekniska Universitet, Luleå, Sweden

**Keywords:** extreme heat, older adult, health inequalities, adaptation, climate change

## Abstract

**Background:**

Extreme hot weather events are happening with increasing frequency, intensity and duration in Hong Kong. Heat stress is related to higher risk of mortality and morbidity, with older adults being particularly vulnerable. It is not clear whether and how the older adults perceive the increasingly hot weather as a health threat, and whether community service providers are aware and prepared for such future climate scenario.

**Methods:**

We conducted semi-structure interviews with 46 older adults, 18 staff members of community service providers and two district councilors of Tai Po, a north-eastern residential district of Hong Kong. Transcribed data were analyzed using thematic analysis until data saturation was reached.

**Results:**

It was agreed upon among the older adult participants that the weather in recent years has become increasingly hot and this led to some health and social problems for them, although some participants perceived that hot weather did not have any impact in their daily lives and they were not vulnerable. The community service providers and district councilors reported that there is a lack of relevant services in the community to support the older adults in hot weather; and there is generally a lack of public education regarding the heat-health issue.

**Conclusions:**

Heatwaves are affecting older adults' health in Hong Kong. Yet, discussions and education effort regarding the heat-health issue in the public domain remain scarce. Multilateral efforts are urgently needed to co-create a heat action plan to improve community awareness and resilience.

## 1. Introduction

Against the backdrop of global climate change, extreme weather events will become more frequent, intense and last longer (1). Luo and Lau (2) showed that in the Southern China region, from 1980's to 2010's, there has been significant increase in the frequency of heat wave (+0.19 events per decade), heat wave days (+2.86 days per decade) and the duration of the longest heat wave (+0.38 days per decade). Locally, the Hong Kong Observatory (HKO) has reported that the numbers of hot night (highest night-time temperature >28°C) and very hot day (highest daytime temperature >33°C) have been respectively on the rising trend. In 2021 alone, Hong Kong has recorded 61 hot nights and 54 very hot days, both hit the record high since 1884 (3). In 2022, the first very hot weather warning (VHWW) in the year was issued on 29 April, 2022, which was a record earliest date for such warning. Moreover, Hong Kong's unique mountainous topography has limited the development of its land, resulting in only around 24% of its territory being developed (4). High-rise buildings are thus concentrated in urban areas; the ventilating airways from the sea to the inland areas are blocked to different extents, exacerbating the urban heat island effect (5).

Extreme hot weather brings negative impacts on people's health and wellbeing (6, 7). In Hong Kong, it has been reported that extreme hot weather events are related to higher risk of adverse health outcomes such as mortality (8, 9), asthma hospitalization (10) acute myocardial infarction among patients with diabetes mellitus (11), all-cause accident and emergency department visits (12) and suicide deaths (13). Moreover, the impact of extreme hot temperature could be more serious for the vulnerable groups in the population. Older adults aged over 65-year-old are more prone to heat-related illnesses than younger age groups (14–16). Their ability of thermoregulation has become less effective following the natural aging process. For example, their ability to control vasodilation to increase blood flow to the skin could be deteriorated. Their decreased sensitivity to thirst could lead to dehydration amid very hot weather. Previous studies have found that the heat-related mortality rate of the older adults has increased globally by more than 53% in the past two decades (17). Several studies projected that the heat-related mortality rate would continue to rise when considering the current rising trends of temperature and aging population (18, 19). In a local study, Wang and colleagues (20) observed 2.53% and 5.33% increases in mortality risk for older adults suffering from a single hot night and five hot nights respectively. When the older adults suffer from two hot days and three hot nights consecutively, their daily mortality risk rises by 5.87%. The older adult's ability to perspire could also be hindered by common medications for chronic diseases such as beta blockers and anticholinergics (21, 22).

Given the health impact brought by extreme hot weather, it is therefore important for individuals to take appropriate adaptive response to minimize the health risks. It was shown that proper actions in response to weather warning might help mitigate the adverse outcomes among the older adults (23). In the health decision-making literature, individuals are often expected to go through cognitive processes that involve weighing risks for consequences against the benefits of taking actions. Grothmann and Patt (24) posited that there are two important cognitive factors involved–risk perception and perceived adaptive capacity. Risk perception measures how an individual perceive the risk of whether a certain event could happen and hence ultimately drives relevant adaptive behaviors. This process involves judging how likely one is exposed to the extreme weather conditions, how harmful such conditions would be to things that one has reason to value (e.g., health), and how one weighs these risks over other priorities in life. This has also been previously theorized in health behavior models that predict behavioral changes, such as the health belief model (25) and the protection motivation theory (26). On the other hand, there are two dimensions for adaptive capacity. First, subjective adaptive capacity concerns how people perceive the resources at their disposal. Gardner and Stern (27) suggested with empirical data that people often perceive little control over global and regional environmental problems. The other dimension concerns, objectively, the social resources available (28). Adger (29) argued that the capacity of individuals to adapt to climate change “is a function of their access to resources,” corroborating that social determinants have a substantial influence on behaviors conducive to health (30, 31). For instance, in the case of the 1995 Chicago heat wave, socioeconomic resources had substantial impact for older adults to adapt to the impact of hot weather, partly through the ownership and utilization of cooling devices (32). A more recent study in Brazil showed that less developed cities showed stronger associations of heatwaves and all-cause hospitalizations (33).

Although older adults are considered as more vulnerable to heat-related illnesses, previous studies showed that older adults or the general population seldom perceived so. In a population-representative cross-sectional survey of older adults in Australia, Hansen and colleagues (34) reported that, when asked whether the older adults concern their health amid a heatwave, only 3 to 6% of the respondents reported that they did. Around 30% said they were not concerned at all in the case when a heatwave is coming. Abrahamson and colleagues (35) conducted semi-structured interviews with 73 older adults in the UK and found that few respondents considered themselves either old or at risk of suffering from the effect of heat; most also claimed that they have taken appropriate steps to mitigate the impact of heat. A recent systematic review by Vu and colleagues (36) echoed that many older people surveyed in Australia, UK, USA and Canada were not aware of their susceptibility to hot weather. In eastern China, a cross-sectional study also reported that the older adults and the lower educated were less likely to perceive hot weather as a health threat (37). A recent population-based telephone survey in Hong Kong showed that about half of around 1,000 respondents recognized that climate change posed as a health risk (38). However, risk perception and behavioral adaptations in the older adults, and the view from frontline service providers, were seldom assessed in the local context. Therefore, in this study, we would like to address the following questions:

- What are the perceptions of the older adults in Hong Kong toward hot weather and what measures are they taking in the face of it?
- What are the current services provided by elderly service providers, particularly the community centers, that targeted at helping the older adults to survive the hot weather?
- What are the possible enablers and barriers and facilitators for those services and measures?

## 2. Materials and methods

### 2.1. Setting

This study consists of two parts. In the first part we examined the perceptions of the older adults toward hot weather and their adaption strategies through focus group discussion. And in the second part, we assessed the perceptions from the point of view of frontline service providers–the problems or barriers they face during heatwaves.

In the first part of the study, older adults, were recruited from two public housing estates of Tai Po district in Hong Kong: Kwong Fuk Estate and Tai Wo Estate. Tai Po is located northeastern of New Territories in Hong Kong. This district has the total population of 316,470, accounting for 4.3% of the total population of Hong Kong. The proportion of the population aged 65 or above is 18.5%, which is similar to the Hong Kong average of 19.6%. Tai Po was one of the districts of the second phase of the New Town development in Hong Kong which started in 1979. This district comprises residential and industrial areas to create a self-contained district. Tai Po is also well-known for its natural landscapes, surrounded by the mountain ranges on the north, west and south, and fronting Tolo Harbor on the east. Kwong Fuk Estate and Tai Wo Estate are two of the eight public housing estates in Tai Po, providing home to some 23,000 population. The former consists of eight residential buildings completed in 1983, while the later consists of twelve residential buildings completed in 1989. Therefore, the two estates were chosen on the basis that they represent typical settings of residential districts of Hong Kong with a wide variety of community facilities having been built in close proximity to the housing estates, ranging from shopping malls, community centers, recreational facilities, and open space.

In the second part, participants were the community service providers and district councilors, who were recruited from the community centers or through bulk email. In Hong Kong, the District Council serves as the interface between the Government and the community and coordinate activities in the provision of services and facilities at the district level. Community support services for the older adults are mainly coordinated under the Social Welfare Department. There are two types of community centers, namely Neighborhood Elderly Center (NEC) and District Elderly Community Center (DECC). Both NEC and DECC provide comprehensive services to facilitate older population to age-in-place, whereas NEC are at the neighborhood level and DECC are at the district level. There are 171 NEC and 41 DECC in Hong Kong as at mid-2022 (39, 40).

### 2.2. Ethics

The study was approved by the Survey and Behavioral Research Ethics Committee of the Chinese University of Hong Kong (ref: SBRE-20-799) and the Institutional Review Board of the University of Hong Kong /Hospital Authority, Hong Kong West Cluster (ref: UW22-304). All participants provided informed consent to participate in the study. Demographic information was collected prior to the start of focus group. Participants were advised that they were free to refuse to answer any question and could withdraw from the study at any time. All identifiers were removed from the transcripts and questionnaires and replaced with pseudonyms.

### 2.3. Participants

We used purposive sampling of participants for the current study with the following selection criteria. Purposive sampling is widely used in qualitative study to recruit information-rich participants who are knowledgeable about the neighborhood and the phenomenon under study (41). The first part of the study included older adults who (1) were aged 60 or above, (2) were Chinese origin, (3) resided in one of the two housing estates (Kwong Fuk Estate and Tai Wo Estate) in Tai Po for at least 1 year, and (4) able to communicate verbally in Cantonese. Recruitment efforts included flyers, referrals from elderly centers, and word of mouth. The second part of the study included service provider or social workers, who had at least 1 year experience working at the NECC or DEC, as well as district councilors in the Tai Po district.

### 2.4. Data collection

There were eleven focus groups, consisting of eight focus groups for older adults and three focus groups for community service providers/district councilors, all of which were conducted in the community centers near the given housing estates between May and July 2021 and online platform in July 2022. Each focus group had 4–8 participants and lasted for approximately 60–90 min. All focus groups were audio recorded and transcribed verbatim.

A semi-structured question guide was created for facilitators to use when conducting focus groups. The focus group interviews with the older adults began with broad questions regarding how participants described hot weather in Hong Kong, the impact of hot weather on their daily lives, the adaption strategies they used under the hot weather and also the factors in the social environment that facilitate or hindered adaption. For the focus group interviews with community service providers/district councilors, we asked whether there have been existing programmes or coordinated community efforts regarding extreme heat, what the way forward is and what potential barriers they foresee.

### 2.5. Data analysis

Data analysis followed a thematic analysis approach involving key stages of organization, reduction and refinement (42). Initially, four researchers independently read each transcript to familiarize with the entire dataset and generated an initial list of codes that were relevant to research aims. These codes were then categorized into the potential themes. Initial codes and the potential themes were discussed among the four researchers in order ensure similarities and to review the emergent themes and refine codes. This process of reviewing themes and recoding data continued until all three researchers reached the agreement regarding the emergent themes and coding schemes. Data saturation was reached when no new themes emerged from the data (43).

## 3. Results

### 3.1. Participants

A total of 46 older adults, 18 staff members of NGOs and 2 Tai Po district councilors joined our focus group interviews. The characteristics of the older adult participants were shown in Table 1.

**Table 1:** Demographic characteristics of the older adult participants (*n* = 46).

**Characteristics**		***n***	**%**
Age	50–59	1	2.2
(mean: 68.61; range 50–85)	60–69	27	58.7
	70–79	15	32.6
	80 or above	3	6.5
Gender	Female	35	76.1
	Male	11	23.9
Education level	Primary or lower	15	32.6
	Secondary	30	65.2
	Tertiary or above	1	2.2
Marital status	Single	2	4.3
	Married	34	73.9
	Widowed	7	15.2
	Divorced/separated	3	6.5
Housing type	Public housing	32	69.6
	Subsidized sales flat	6	13.0
	Private permanent housing	8	17.4
Number of years living in Tai Po	< 10 years	2	4.3
	10–19 years	4	8.7
	20–29 years	5	10.9
	30–39 years	32	69.6
	40 years or above	3	6.5
Living arrangement	Living with spouse only	22	47.8
	Living with children only	2	4.3
	Living with spouse and children	11	23.9
	Living alone	10	21.7
	Living with others (e.g., domestic helper)	1	2.2
Employment	Retired	41	89.1
	Homemaker	4	8.7
	Unemployed	1	2.2
Monthly personal income^#^	< HK$2,000	20	43.5
	HK$2,000–3,999	16	34.8
	HK$4,000–9,999	6	13.0
	HK$10,000 or above	3	6.5
	Refuse	1	2.2
CSSA holder*	Yes	2	4.3
	No	44	95.7
Perceived financial sufficiency	Insufficient	9	19.6
	Tight/enough	32	69.6
	More than enough	4	8.7
	Very sufficient	1	2.2
Self-rated health	Poor or fair	28	60.9
	Excellent, very good or good	18	39.1

# HKD$1 = USD$0.13.

* CSSA: comprehensive social security assistance (a social welfare in Hong Kong that provides supplementary payments to residents who are not able to sustain themselves financially).

### 3.2. Key findings

We grouped findings from all participants into five main themes: (1) Perceived impact of hot weather, (2) Adaptation appraisal, (3) Enabling social environment, (4) Perceived barriers to adaptation to hot weather, and (5) new services that could be implemented. We presented the illustrative quotes for each theme in Table 2.

**Table 2:** Illustrative quotes for each theme.

**Quote number**	**Quote**	Participant characteristics*
**Theme 1: Perceived impact of hot weather**		
**Theme 1.1: Changing pattern of hot weather**		
Q1	“*Compare to the old-time, the weather condition now is not as cool, nor is the temperature at night. It's not comfortable… when I work in the daytime, it is tough, and I can't stop sweating.”*	W 70
Q2	“*It's hot, very hot (all)… especially the recent hot weather… the hot weather came earlier than before… it used to come in June or July (now it's not yet mid-May)”*	W 65 and W 74
Theme 1.2: Physical health		
Q3	*(Interviewer: will you suffer from insomnia?) “Yes, I will. Since my apartment faces the West, the sun heats up my room very seriously during the daytime. We need to push back our bedtime until late at night, so we can save the spending on electricity, just turn on the fan to cool off the room. If it's too hot, we still turn on the air conditioner.”*	W 50
Q4	“*When I am hot, my face feels like boiling, I cannot breathe normally and my heart beats very quickly.”*	W 65
Q5	“*Especially walking with the mask on, I have difficulty breathing and get dizzy so easily.”*	W 64
Q6	“*We collaborated with Personal Emergency Link Service; they informed us whenever there were seniors who pressed the emergency alarm… Sometime later we sensed that there seemed to be a trend, although we never really analyzed from data. We think [more seniors] reached out to seek help in heatwaves.”*	M [DECC worker, exp 10 years]
Theme 1.3: Mental health		
Q7	“*Sometimes the hot sunlight annoys me, especially the air is still, I become impatience and easily irritated under this kind of weather.”*	W 69
Q8	“*For example, when the weather is hot, our mood can be affected easily. When you get irritated, your blood pressure would go up… people can get irritated easily when feeling hot in the hot weather.”*	W 63
Theme 1.4: Reduced social activities		
Q9	“*I will not leave home often during the summer, won't do it unless necessary.”*	W 70
Q10	“*It must be (affecting me to see my friends or family). I won't leave home unless it's necessary, don't you think so? It's too hot.”*	W 65
Q11	“*(Normally,) I will walk for an hour. If the weather today is as hot as yesterday, I will only walk for half an hour.”*	M 72
Theme 1.5: Not concerned about hot weather		
Q12	“*I don't always turn on the electric fan, just use a handheld fan to cool down. I feel all right, not too hot. I seldom turn on the electric fan… I don't think it is that hot.”*	W 80
Q13	‘*Some elderly think they will catch a cold easily under the cold weather. However, when the weather is hot, it is unlikely to cause death or harm to their body… not as relevant compared to the cold weather, based on their experiences.'*	M [DC, exp 1 year]
Q14	“*I visited a client yesterday. I sweated profusely but my client looked calm. When he saw me wiped the sweat off, he asked, “Do I need to turn on the fan for you?”.”*	M [DECC worker, exp 12 years]
**Theme 2: Adaptation appraisal**		
Theme 2.1: Indoor strategies		
Q15	“*When it is hot, I will turn on the air conditioning or electric fan… or take a shower… Usually, I will turn on the air conditioning at night and use the electric fan in the afternoon.”*	W 63
Q16	“*The best way to do it is to open the windows and doors, not turn on the air conditioner… we always open the windows… when the windows are opened, the breeze can come into the apartment by the convection currents.”*	W 70
Q17	“*I will take a shower twice, one in the morning, and another one at night. If it's too hot, I will take a third shower.”*	W 68
Q18	“*We drink plenty of water during the summer.”*	W 74
Theme 2.2: Outdoor strategies		
Q19	“*If I am going to do exercise in the morning, I will go early to avoid the hot sunlight, so not as hot.”*	W 65
Q20	“*If the weather is hot, I will go out once in the afternoon and go to the supermarket to enjoy the air conditioning. Then, I will walk to other places and rest in between when the weather is too hot.”*	M 61
Q21	“*Inside my backpack, there are a bottle of water, a parasol, a fan, a towel and miscellany. I always use the parasol when I walk and wipe away my sweat with the towel.”*	W 67
Q22	“*They will hang around Wan Tau Tong Shopping Center and Uptown Plaza. There are plenty of seats and usually [the older adults] won't get driven out. But some older adults will spend the entire day there, from the morning to the evening… some older adults will come to our center and community halls.”*	W [NGO worker B, exp years not available]
**Theme 3: Enabling physical and social environment**		
Theme 3.1: Accessible public space with cooling features		
Q23	“*There is a banyan tree shade (at the park). The banyan tree has been there for a long time and is well-taken care of with pruning. It is cool, we do exercise over there as there are tree shade and a gazebo next to it. When it is raining, we will stand under the gazebo to shelter from the rain.”*	W 67
Q24	“*The facilities are pretty good over there (Mui Shue Hang). It is a shaded area in the afternoon, no direct sunlight.”*	M 67
Theme 3.2: Housing design		
Q25	“*My apartment is inside a hash-shaped building. It's comfortable, won't get too hot during summer and not too cold during winter. The apartment is faced to the southeast and is right across from the community hall. I have never turned on the air conditioner.”*	W 84
Q26	“*Since I live in the corner flat inside a Y-shaped building (trident), I have extra three windows, so when I open the doors and the windows… because my flat is at the end of the corridor, the breeze can reach my flat through convection currents, so it is not very hot.”*	W 63
Q27	“*Y-shape building, it is hard for the breeze to reach the center of the building.”*	M [DC, exp 1 year]
Q28	“*When I stay home during the daytime, I usually don't turn on the electric fan. It is because my apartment is faced to the southwest, when I sit next to the window, I can feel the breeze coming through the window. It's quite comfortable.”*	W 67
Q29	“*There is nothing I can do. Our apartment is faced to the west, the sun starts to shine on the apartment at noon until 5–6 pm. It is quite suffering… can't avoid the heat from the sun living in an apartment facing west.”*	W 65
Theme 3.3: Channels to access hot weather information		
Q30	“*Something was shown on the television that said if a person stays under the sun for too long, it may cause dizziness and fall. It reminds people to be alerted of the hot weather today.”*	W 70
Q31	“*From the television. There is a weather forecast on television that shows the temperature during the day and night. The weather forecast by the Hong Kong Observatory is quite accurate… it's pretty accurate, over 90% accuracy… won't say it is 100% accurate.”*	W 68
**Theme 4: Perceived barriers to adaptation to hot weather**		
Theme 4.1: Economic concern		
Q32	“*We try to push back the time to turn on the air conditioner. For example, we will turn on the air conditioner when we are ready for bed. We won't turn it on beforehand because the cost of electricity is expensive.”*	W 69
Q33	“*It is wasteful to turn on the air conditioner just for one person.”*	W 65
Q34	“*I have come across clients who lived in squatters which was built from zinc and iron sheets. You could not imagine how they fare. It was hot outside – some 30 degrees, but even hotter inside – around 40 degrees. There were older adults who live in these kinds of places alone.”*	M [DECC worker, exp 10 years]
Theme 4.2: Perspectives from Chinese medicine or general dislike of air conditioning		
Q35	“*When we go to see a doctor, especially in the perspective of Chinese medicine, they say it is not healthy to stay in an air-conditioned environment for too long. It may increase the risk of getting arthritis. So, it is better to avoid staying in that environment for too long. Even if you turn on the air conditioner to sleep, you will feel more tired the next morning.”*	W 69
Q36	“*Elderly people believe in nature, or nature in the perspective of Chinese medicine. They believe the cool air from the air conditioner is not good for their health.”*	M [DC, exp 1 year]
Q37	“*Older adults usually like to sit at the parks and socialize. I've once invited them to come to the community center because it was hot outside. But they seemed not interested in getting rest under air conditioning. They rather chose the park.”*	M [NEC worker, exp 1.5 years]
Theme 4.3: Outdoor space not conducive to cooling		
Q38	“*So, I always stay home (when the weather is hot) … because it is too hot outside. There are many places without cover.”*	W 69
Q39	“*The shopping malls here are too small to walk around… But the malls don't provide seats as well… you can only walk or stand.”*	W 62
Q40	“*Before the renovation of the Tai Wo Market, there were seats provided for people to rest. Now, the seats are removed after the renovation and due to infection control. There are not many places left for them to hang out, only at the restaurant, but can't stay long.”*	W [NGO worker A, exp years not available]
Theme 4.4: Lack of discussion in the public discourse		
Q41	“*I really don't know (there are heat shelters nearby), even though I have been living here for a long time.”*	W 65
Q42	“*Under the extremely hot weather, I believe no one goes to the heat shelter. Elderly people don't prefer to go there to rest because there is nothing inside. How do you ask people to stay there?”*	W [NGO worker C, exp years not available]
Q43	“*I think the news has reported heat-related issues, but not much about how hot weather could negatively affect our bodies. So, elderly people are not aware of this issue.”*	W [NGO worker C, exp years not available]
**Theme 5: New services that could be implemented**		
Q44	“*Since last year, we have been planning some pool activities for our clients in the community swimming pools – it was fun for cooling.”*	W [DECC worker, exp 12 years]
Q45	“*Our community center this year also plan for opening also during weekends and public holidays… so that they could come hang around and enjoy air conditioning, or they could enjoy intergenerational activities with their grandchildren”*	M [NEC worker, exp 3.5 years]
Q46	“*As we have experienced the pandemic of COVID-19, we would notify the members through WhatsApp, which is widely used nowadays, about 60% of the members are covered and they are used to receive our message. This could serve as an alarm system for heatwaves in the future.”*	W [DECC worker, exp 12 years]

* Men (M)/Women (W); numbers are age in years unless otherwise specified; district councilor (DC); worker from district elderly community centers (DECC)/neighborhood elderly centers (NEC)/non-government organization (NGO); experience (exp) in years.

#### 3.2.1. Theme 1: Perceived impact of hot weather

##### Theme 1.1–Changing pattern of hot weather

It was generally agreed among the participants that summers in Hong Kong are becoming increasingly hotter and arrive significantly earlier than one or two decades ago (Quote (Q) 1 and Q2, Table 2).

##### Theme 1.2–Physical health

Some of the older adults expressed that the increasingly hot weather is affecting their physical health. Symptoms such as poorer sleep quality, headache, dizziness and racing heart were sometimes experienced when they went outdoor during summertime (Q3 to Q6). Notably, universal masking intervention during the COVID-19 pandemic has exacerbated the discomfort brought by extreme heat (Q5).

##### Theme 1.3–Mental health

Hot weather also affects mental health–some of the participants reported that they feel more irritable when the temperature is hot (Q7 and Q8).

##### Theme 1.4–Reduced social activities

Some participants reported that they inclined to go out less to avoid heat in hot summer. In such a way, they felt that this situation reduced socialization with friends and relatives (Q10) and physical activities in the outdoor areas (Q9 and Q11).

##### Theme 1.5–Not concerned about hot weather

However, some participants felt that the increasingly hot weather does not have much impact on their health or daily activities (Q12). A district councilor quoted from the older adults whom he came across in his duties, saying that they felt the cold in winter could lead to respiratory diseases, while the heat in summer is less relevant to health (Q13). Another community worker reported an episode of home visit in a hot summer, and his client did not seem to be bothered at all (Q14).

#### 3.2.2. Theme 2: Adaptation appraisal

For the older adults who found that the increasingly hot temperature bothers them during summertime, they came up with various solutions to deal with the situation. Here, we grouped the solutions into indoor and outdoor ones. Generally, the participants were aware that there are strategies that they can adopt to stay away from heat and staying indoor during extreme heat is usually more advisable.

##### Theme 2.1–Indoor strategies

When the participants stayed home, they would employ strategies that could improve ventilation, such as opening the windows and turning on fans (Q15 and Q16). They would also use methods to cool themselves directly, such as bathing (Q17). Some participants suggested that they increased hydration during the heat (Q18). Air conditioners have been mentioned as a way of cooling, despite the older adults used it more sparingly (discussed more in Theme 4).

##### Theme 2.2–Outdoor strategies

When the participants needed to go outside, some of them reported that they make plans to avoid the heat. For instance, they chose to go out at a time of the day when the heat is less intense, i.e., early mornings or late evenings (Q19). They would reduce the number of times that they need to go out by, say, group several days' of grocery shopping in 1 day. Some of the participants were adept at planning routes between two points through which they could enjoy shaded walkways (Q20). They also plan what to pack in their bags during extreme heat, mainly portable fans, umbrella, towels and a bottle of water (Q21). Despite of the measures they prepare for outdoors, a staff member of NGO reported that some older adults whom they serve preferred to stay in air-conditioned malls during most of the daytime in summer, while some of them would prefer to go to local community centers (Q22).

#### 3.2.3. Theme 3: Enabling physical and social environment

##### Theme 3.1–Accessible public space with cooling features

Participants were generally satisfied with the outdoor environment of the Tai Po district, in particular the parks and greenery that are available to them to seek shelter from heat where there is good ventilation (Q23 and Q24).

##### Theme 3.2–Housing design

Views varied on the design of the buildings in which the live. Some opined that the so-called “hash-shaped” (it is actually a double-tower design resembling two hollow squares joining at one corner of each square) design of their building facilitated ventilation such their homes are much cooling than outside (Q25). Participants who resided in Y-shaped towers, which have semi-enclosed public areas inside buildings, had mixed opinions–some said they feel good ventilation while some disagreed (Q26 and Q27). Participants also agreed that the orientation of the buildings matter for good ventilation because directly facing the sunlight during the day builds up heat in the flat (Q28 and Q29) (Photos of the façade of the buildings in Supplementary material).

##### Theme 3.3–Channels to access hot weather information

Regarding information about hot weather in the public domain, the participants were aware of the hot weather warning issued by the HKO. Most of them received the information regarding hot weather from radio and television channels (Q30 and Q31) and they are generally satisfied with information about hot weather in the public domain.

#### 3.2.4. Theme 4: Perceived barriers to adaptation to hot weather

##### Theme 4.1–Economic concern

The opinions on the use of air conditioners as a cooling strategy at home have been mixed. On one hand, the participants agreed that air conditioners are indispensable tools that bring optimum thermal comfort; on the other hand, the costs for electricity have been a concern for most of them given they were all retired with very limited income. Consequently, they adopted various other strategies to limit its use. For example, they only use air conditioners when most of the household members are present, a way what they claimed to be more “cost-efficient” (Q32 and Q33). To the more extreme end, some frontline workers opined that they came across cases in much worse housing conditions with older adults living alone faring poorly in hot weather since they could not afford to rent more proper housing (Q34).

##### Theme 4.2–Perspectives from Chinese medicine or general dislike of air conditioning

Interestingly, some older adult participants perceived that the “cold” and “damp” from the air conditioners is not good for their health from the Chinese medicine perspective, which could make them feel more tired and musculoskeletal pain (Q35 and Q36). If they were to choose, sometimes they would prefer natural wind rather than air conditioners. A community frontline worker said that his clients rather enjoy the breeze in the park rather than air conditioning in the community center (Q37).

##### Theme 4.3–Outdoor space not conducive to cooling

The participants opined that there are not adequate shading devices in their neighborhood, especially in places which they frequently go, such as the bus stops and podium (Q38). Even though there are air-conditioned malls in the neighborhood, the older adults could hardly find a place to sit and relax inside the malls (Q39 and Q40). It appeared that malls generally do not seem to welcome older adults merely sit in the malls and seek shelter from heat (Q22).

##### Theme 4.4–Lack of discussion in the public discourse

The increasingly hot weather is giving, to some extent, more hassles to the older adults in their daily lives. However, the impact of heat is seldom discussed in any public discourse regarding what proper actions to take in the face of more frequent and intense extreme heat. The participants generally did not perceive the government has done adequately to address the problem of extreme heat. Although the government opens the community heat shelters at night when VHWW is hoisted, most of the participants expressed that they were not aware of this at all. Some frontline NGO workers also felt that the community heat shelters were not attractive for the older adults, and that discussions of the heat-health issue were almost non-existent in the public domain (Q41 to Q43).

#### 3.2.5. Theme 5: New services that could be implemented

Service providers generally agreed that as compared to services targeting at the cold weather, there were sparse services targeting at the hot weather. Even though there are still regular services provided by the NEC/DECC such as home visits and community health talks, the activities were not designed to heed to the health needs of the older adults in hot weather. The frontline service workers have brainstormed some forms of activities for future considerations. One participant mentioned a water exercise group in their community center to encourage older adults to maintain physical activity during summer, but that was suspended due to the pandemic (Q44). One service provider shared their plan to open the community centers on Sunday and public holidays. In this way, the older adults could have intergeneration activities and at the same time having a cool shelter (Q45). Another frontline worker also suggested that one could send picture reminders to their clients via texting apps such as WhatsApp to remind them to take appropriate precautions during hot weather (Q46). Taken together, solutions that are creative and without additional manpower and financial resources are urgently warranted.

## 4. Discussion

### 4.1. Summary of findings

This study provides insights into the perception and awareness of extreme hot weather among the older adults in a north-eastern neighborhood in Hong Kong and how these older adults adapt to the hot weather and the possible barriers that they face. It was agreed upon among the participants that the weather in recent years has become increasingly hot and this led to some health and social problems for them, although some participants perceived that hot weather did not have any impact in their daily lives and they were not vulnerable. Despite the seriousness, the heat-health issues so far have not received proportionate attention in the public domain. Public education in this regard has been sparse. From the service providers' point of view, there is also a lack of relevant community services or support for older adults in hot weather. However, the older adults we interviewed were generally flexible and adapting to the increasingly hot weather with their own means, albeit their conception about air-conditioners from the Chinese medicine point of view might have limited their use. Indoor environment and community facilities are important resources for older adults to adapt to hot weather.

### 4.2. Comparison with previous studies

Our findings that some older adults perceived that hot weather did not constitute a health concern and that they are not vulnerable groups are consistent with previous findings in Western settings (35, 44). There could be two reasons for this finding. First, understandably, some participants noted that they endured intense manual labor jobs in the pre-air-conditioned era, further strengthening the belief that they were well in control of their health and adaptation was not necessarily. Second, older adults might be less likely to adopt adaptive behaviors given their physiological decline following the natural process of aging such that they become less sensitive to ambient temperature and thirst (14). We also highlighted that extreme hot weather affects older adults' mental health when some participants reported that they felt more anxious when the weather is hot and such weather conditions limited their social and physical activities, which was similar to what was reported in a previous study in Adelaide of Australia (45).

Overall, the older adults who we interviewed mostly agreed that the changing climate increasingly become a problem to them, and they were reasonably flexible to adapt. A previous local cross-sectional study reported that perception of risk of extreme hot weather is not related to the utilization and ownership of cooling devices in the Hong Kong population (38). Our study corroborated by showing that almost all the older participants we interviewed owned air-conditioners, but they are more reluctant to use them. Although the use of air-conditioners has been considered as one of the strong protective factors against heat-related illnesses (46), its use could be limited especially in older adults given the concern for the cost of electricity, a finding which is consistent with a previous qualitative study conducted in a sample of older adults in Australia (47). This is perhaps partly reflective of the relatively lower socioeconomic status of our participants, who mainly lived in public housing estates, compared to the rest of the Hong Kong population.

We found that our participants often reported that they reduce the frequency of going out when the outdoor temperature, as informed by the media, is hot. This is opposite to what was reported in a small study with 29 older adult households of Detroit of the United States (48). Using hourly logs to record the participants' behaviors, White-Newsome and colleagues (48) reported that the older adults were more like to go outside of their places of residence when outdoor temperature increased. This is perhaps because the building density of Detroit is less than that of Hong Kong, resulting in a less intense urban heat island and greater thermal comfort even amidst the heat. Reduced ventilation in typical residential neighborhoods in Hong Kong also discourages outdoor activities.

### 4.3. Policy implications

Heat action plans are now common in many developed countries (21, 49). However, the perspectives of public health were not integrated in the current climate action plan even given mounting local and international evidence of the heat-health relationship. Currently, the HKO issues VHWW signal to alert the public and, accordingly, the Home Affairs Department operates temporary night-time heat shelters when such warning is hoisted. The night-time heat shelters only seemed to have lukewarm reception in the public because they are usually situated not according to the geographical distribution of urban heat hazard spots (50) and there are no meaningful activities to engage their users, discounting the usefulness and attractiveness of these centers. It is welcome to see that, as of recently, the HKO added messages along with the VHWW signal to remind the public to take particular care of older adults and other vulnerable groups in the case of heat. However, other than that, as noted by our community service providers in this study, the discussion of the heat-health issue has only very recently started to gain traction in the public discourse. The Hong Kong government recently published the Climate Action Plan 2050, which proposed that as a part of the plan to mitigate urban heat, the government will work to improve building design and increase urban vegetation. However, little was discussed on how to prepare the local community to become more aware and prepared for the future climate scenario. Resources are often available in the community, but there is a lack of ingenuity and collective will to integrate and coordinate these resources into helping especially the vulnerable groups to adapt to extreme hot weather. For instance, several of our older adults participants and community service providers opined that publicly air-conditioned spaces in Hong Kong such as shopping malls can provide a cool environment for the older adults without the worry of costs of electricity. Unlike in other settings, these public spaces in Hong Kong are often within walkable distances within a community hence the older adults can reach easily (47). Nonetheless, it was mostly agreed upon by our participants that these spaces often lack places for older adults to sit and relax.

Contextualizing the heat-health issue in the older population entails an understanding of the underlying social, cultural, and institutional factors. It is therefore context-specific for the older adults in Hong Kong to believe that the use of air-conditioners has implications to their health from the angle of Chinese medicine, as discussed by some of our participants. Such aversion could have stemmed from the idea that “nefarious Wind” could cause “disharmony” in the body and causes symptoms generally called “external wind cold” such as headaches, generalized aches and runny nose (51). While part of it could be related to influenza-like illnesses, this conception could affect older adults' attitude to use air-conditioners. It is therefore suggested that when formulating heat-health messages to the older adults in Hong Kong or more broadly the Chinese community, a wide variety of alternative strategies could be suggested in addition to the use of air-conditioners such that older adults could be provided with ample choices of strategies to adapt.

In addition to public air-conditioned spaces, as our participants noted, another indoor space that was deemed to be an important shelter from heat is their homes. Compact living spaces in high-rise buildings is one of the unique characteristics of urban configuration of Hong Kong, which often linked to poor thermal comfort resulting from intense solar radiation, poor ventilation, and the slow release of heat from building materials, particularly during intense heat in summertime (52). As a matter of fact, air conditioning at home still remains a major solution for older adults to relieve from heat. It is welcome to see local charities started community initiatives to subsidize electricity bills for older adults who are financially incapable (53). The government could also consider the possibility of formal subsidy for electricity for needy older adults during hot seasons or minor home modifications to improve indoor thermal comfort. However, in the long run, our city has to adopt of adaptive design following the future climate scenario. Strategies such as using higher albedo materials covering urban built surfaces (e.g., building ceilings or pavements), facilitation of air turbulence within a community or incorporation of blue-green spaces were all proven to bring better thermal comfort for urban dwellers (54). The use of air-conditioning could therefore be minimized.

Our interviews revealed that the community service providers were in general not well equipped with heat-health knowledge. This is in line with the lack of public discourse and could potentially explain a lack of coordinated and targeted efforts to mitigate the heat situation faced by the older adults. Empowering the frontline workers of DECC and NEC in Hong Kong as well as the district councilors will constitute a salient strategy as they have expansive reach to older adults in the community. Reaching out to particularly vulnerable targets, such as those living in inadequate housing, living alone or those with dementia, will be essential. Creative strategies such as texting picture reminders to their clients will also be potentially helpful.

### 4.4. Strengths and limitations

The current study has recruited both older adults and frontline workers in the community, who have enriched our insights from the angle of service providers in addition to the subjective experience of the older adults. Our interviews were conducted in summertime such that the participants did not have to rely on memory to recall the experience of hot weather. However, the current study has limitations. First, our older adult participants were mostly sampled from the two designated public housing estates in Tai Po district. Our sample and hence their opinions might not be representative for older adults in other housing types and from other districts. Future studies could consider recruiting samples from other districts in Hong Kong to confirm the findings in this study. However, in a broader sense, this sample of participants were knowledgeable about the climatic conditions on a neighborhood scale. The meanings and processes of everyday lives in the midst of extreme hot weather expressed by the participants would therefore be relevant to other highly dense urbanized settings. Second, the convenience sampling nature of our study implied that our sample was only constituted of subjects who were more aware of the issue of heat than the general population. Future quantitative studies, such as a territory-wide questionnaire, could confirm the extent to which the older population are aware of the heat-health issue. Third, our sample was only limited to those who were able to independently walk to the community center, which rendered us unable to assess the views of those most vulnerable to heat.

## 5. Conclusions

Our findings showed that older adults in Hong Kong are concerned that extreme hot weather constituted a surging problem for them physically, mentally and socially. While some of the older adults we interviewed were reasonably flexible and adaptive, some others believe that there is no need to adapt, which could be problematic in the long run as the future climate scenario continues to unfold. Discussions and education effort regarding the heat-health issue in the public domain remain scarce. Multilateral efforts are urgently needed to co-create a heat action plan to improve community awareness and resilience.

## Data availability statement

The raw data supporting the conclusions of this article will be made available by the authors, without undue reservation.

## Ethics statement

The studies involving human participants were reviewed and approved by Survey and Behavioral Research Ethics Committee of the Chinese University of Hong Kong. The patients/participants provided their written informed consent to participate in this study.

## Author contributions

EL, KL, and JW conceptualized the study. EL, PC, and KL coordinated the recruitment of subjects and supervised the data transcription process by research assistants. EL, PC, KC, and MK conducted the data analysis, synthesized the findings, reviewed the literature, and wrote the manuscript. All authors participated in critically reviewing and intellectually input to the drafts of the manuscript. All authors contributed to the article and approved the submitted version.
