# Supplementary material for: Perception of extreme hot weather and the corresponding adaptations among older adults and service providers–A qualitative study in Hong Kong
Source: Front Public Health. 2023 Feb 10;11:1056800. doi: 10.3389/fpubh.2023.1056800 (PMC9980346; doi:10.3389/fpubh.2023.1056800)
Supplement: Supplementary file 1 [file Data_Sheet_1.docx]

Supplementary Material

# Supplementary Figures


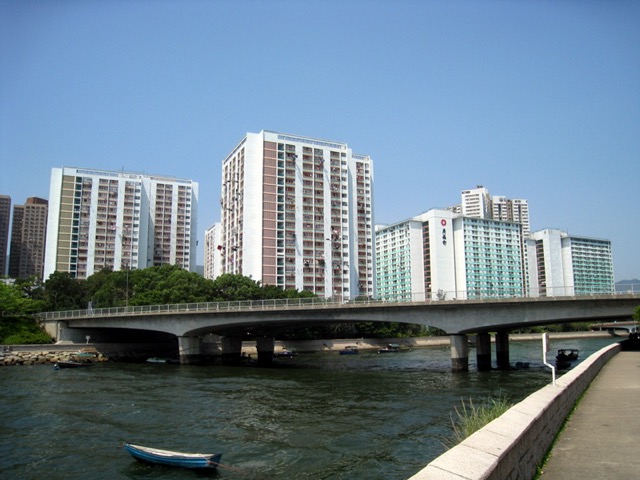


**Supplementary Figure 1.** Double tower building design seen in Kwong Fuk estate, Tai Po, Hong Kong


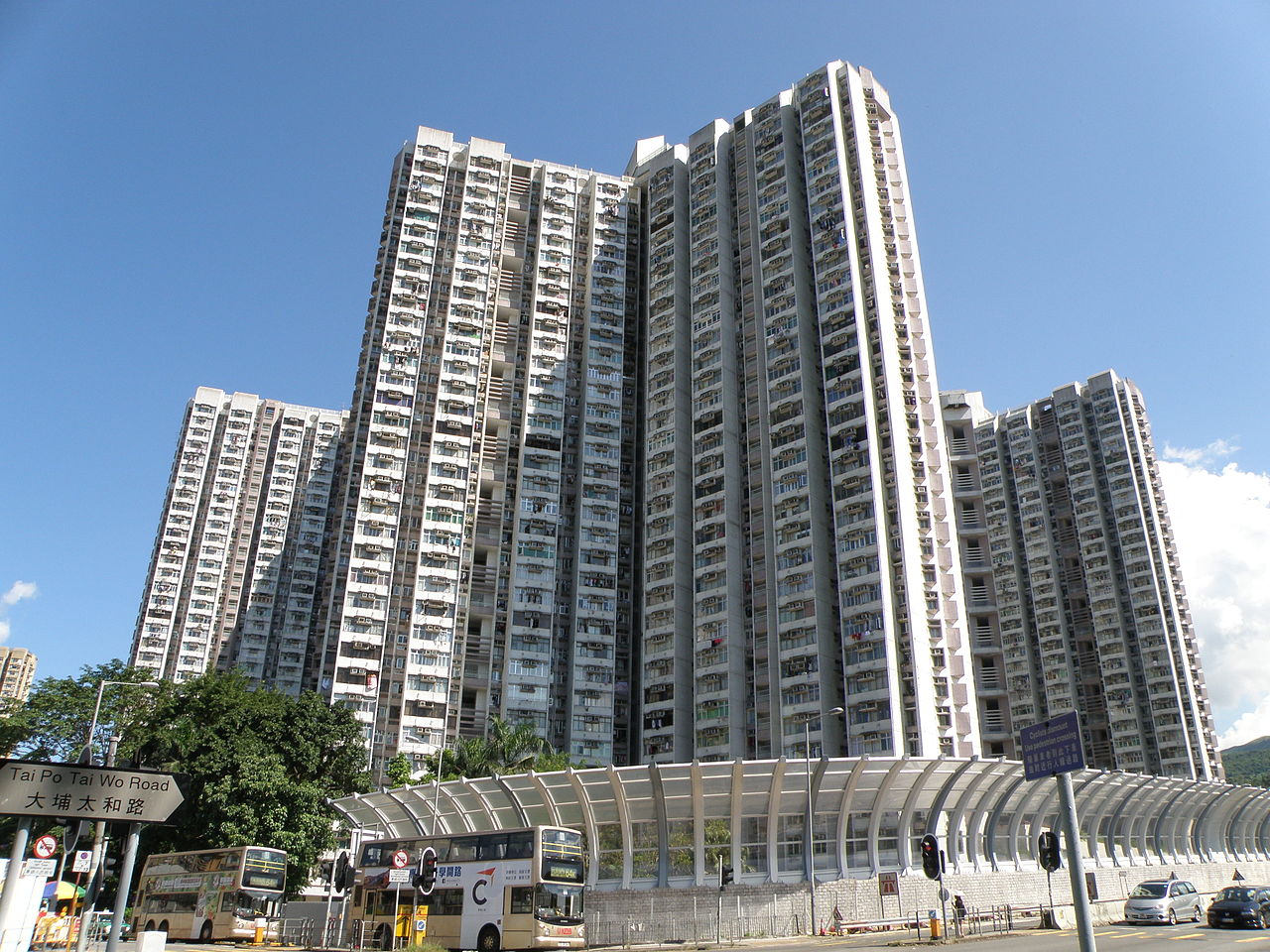


**Supplementary Figure 2.** Y-shaped building design seen in Tai Wo estate, Tai Po, Hong Kong
